# Differential loss of thalamostriatal and corticostriatal input to striatal projection neuron types prior to overt motor symptoms in the Q140 knock-in mouse model of Huntington's disease

**DOI:** 10.3389/fnsys.2014.00198

**Published:** 2014-10-15

**Authors:** Yun-Ping Deng, Ting Wong, Jim Y. Wan, Anton Reiner

**Affiliations:** ^1^Department of Anatomy and Neurobiology, The University of Tennessee Health Science CenterMemphis, TN, USA; ^2^Department of Preventive Medicine, The University of Tennessee Health Science CenterMemphis, TN, USA

**Keywords:** Huntington's disease, corticostriatal, thalamostriatal, premanifest, pathology

## Abstract

Motor slowing and forebrain white matter loss have been reported in premanifest Huntington's disease (HD) prior to substantial striatal neuron loss. These findings raise the possibility that early motor defects in HD may be related to loss of excitatory input to striatum. In a prior study, we showed that in the heterozygous Q140 knock-in mouse model of HD that loss of thalamostriatal axospinous terminals is evident by 4 months, and loss of corticostriatal axospinous terminals is evident at 12 months, before striatal projection neuron pathology. In the present study, we specifically characterized the loss of thalamostriatal and corticostriatal terminals on direct (dSPN) and indirect (iSPN) pathway striatal projection neurons, using immunolabeling to identify thalamostriatal (VGLUT2+) and corticostriatal (VGLUT1+) axospinous terminals, and D1 receptor immunolabeling to distinguish dSPN (D1+) and iSPN (D1−) synaptic targets. We found that the loss of corticostriatal terminals at 12 months of age was preferential for D1+ spines, and especially involved smaller terminals, presumptively of the intratelencephalically projecting (IT) type. By contrast, indirect pathway D1− spines showed little loss of axospinous terminals at the same age. Thalamostriatal terminal loss was comparable for D1+ and D1− spines at both 4 and 12 months. Regression analysis showed that the loss of VGLUT1+ terminals on D1+ spines was correlated with a slight decline in open field motor parameters at 12 months. Our overall results raise the possibility that differential thalamic and cortical input loss to SPNs is an early event in human HD, with cortical loss to dSPNs in particular contributing to premanifest motor slowing.

## Introduction

Premanifest HD individuals are slowed in initiation and/or execution of motor tasks (Siemers et al., 1996; de Boo et al., 1997; Kirkwood et al., 1999, 2000; Blekher et al., 2004; Rao et al., 2008, 2011; Biglan et al., 2009; Bechtel et al., 2010; Delval et al., 2011; Tabrizi et al., 2011; Turner et al., 2011). This defect is mild in individuals not near clinical onset, but more severe in those near onset (Kirkwood et al., 2000; Rao et al., 2008; Bechtel et al., 2010; Rupp et al., 2010). Motor symptoms in premanifest HD occur in parallel with gradual loss of cerebral and striatal white matter (Kipps et al., 2005; Reading et al., 2005; Ciarmiello et al., 2006; Paulsen et al., 2006; Rosas et al., 2006; Hobbs et al., 2010a; Aylward et al., 2011; Dumas et al., 2012), increasing striatal hypometabolism (Grafton et al., 1992; Ciarmiello et al., 2006), and reduced striatal activation during behavioral tasks (Paulsen et al., 2004; Wolf et al., 2012). Nonetheless, the few neuropathological studies of premanifest striatum have reported some variable neuronal loss in the head of the dorsal caudate, but little or no neuron loss has been described for the motor striatum (Vonsattel et al., 1985; Albin et al., 1991; Vonsattel and DiFiglia, 1998).

In a prior study, we examined thalamostriatal and corticostriatal input loss over the first year of life in a precise genetic mimic of human HD, the heterozygous Q140 knock-in mouse (Deng et al., 2013). Heterozygous Q140 mice are not yet overtly symptomatic at 1 year of age (and thus comparable to premanifest human HD) and show no striatal neuron loss (Rising et al., 2011; Deng et al., 2013). We found significant deficiencies in thalamic input to the spines and dendrites of striatal neurons by 4 months of age, and substantial loss of cortical input to the spines of striatal neurons by 1 year. These findings suggest that loss of thalamostriatal and corticostriatal terminals may contribute to motor impairments in premanifest HD.

In symptomatic HD, differential loss of direct pathway striatal projection neurons (dSPNs) vs. indirect pathway striatal projection neurons (iSPNs) occurs and accounts for the differing clinical symptoms at different stages of progression (Reiner et al., 1988; Richfield et al., 1995; Glass et al., 2000; Deng et al., 2004). The differential striatal projection neuron (SPN) loss raises the possibility that any premanifest loss of corticostriatal and/or thalamostriatal terminals from SPNs in human HD, as we had shown in Deng et al. (2013) for heterozygous Q140 mice, may be differential as well. In the present study, we characterized the loss of thalamostriatal and corticostriatal axospinous terminals from dSPNs and iSPNs in heterozygous Q140 mice. The loss of corticostriatal terminals at 12 months was preferentially for dSPN spines, and was correlated with a slight decrease in locomotor activity, consistent with the role of dSPNs neurons in promoting movement and in cortex in driving their activity (Albin et al., 1989; Kravitz et al., 2010; Spigolon et al., 2013). Thalamostriatal terminal loss was comparable for D1+ and D1− spines at both 4 and 12 months of age. The results suggest that an early non-differential deficiency in thalamic input to SPNs, and a later specific loss of cortical input to dSPNs may occur and contribute to premanifest HD motor abnormalities.

## Materials and methods

### Animals

Results from 10 wild-type male (WT) and 10 heterozygous male Q140 mice (obtained from JAX, Bar Harbor, Maine) are presented here, and all animal use was carried out in accordance with the National Institutes of Health Guide for Care and Use of Laboratory Animals, Society for Neuroscience Guidelines, and University of Tennessee Health Science Center Guidelines. Heterozygous HD mutants were studied because the human disease most commonly occurs due to a single allelic defect. It is also important to emphasize that most prior behavioral and histological work on Q140 mice has focused on homozygous mutants (Menalled et al., 2003; Hickey et al., 2008, 2012; Lerner et al., 2012), but one recent study has shown that the heterozygous Q140 phenotype is milder than that in homozygous Q140 mice (Rising et al., 2011). Moreover, Rising et al. (2011) did not find evidence of early hyperactivity in a rearing test at 2.5 months in either heterozygous or homozygous Q140 mice, in contrast to Menalled et al. (2003), who reported rearing and open field hyperactivity at 1 month in homozygous Q140 mice. Thus, the occurrence of an early hyperactivity in heterozygous Q140 mice has neither been shown nor disproven. Because deficiencies in the thalamostriatal projection were evident at 4 months, but loss of corticostriatal input was not evident until 12 months in our prior single-labeling study (Deng et al., 2013), in the present study VGLUT2/D1 double-labeling was analyzed for both 4 and 12 month-old Q140 and WT mice, while VGLUT1/D1 double-labeling was only assessed for 12 month-old Q140 and WT mice. As in prior studies by us and others, we used D1 immunolabeling to distinguish dSPN spines and dendrites (D1–positive) from iSPN spines and dendrites (D1–negative) (Day et al., 2006; Lei et al., 2013). It should be noted that the repeat length in the Q140 mice we used had undergone a spontaneous reduction during breeding at JAX, and the average CAG repeat length in our five 4-month old Q140 mice was 128.6 ± 1.4, and our five 12-month old Q140 mice was 135.0 ± 0.9. The range of CAG variation for Q140 mice within each age group was small and had no significant effect on the outcomes measured here, as assessed by regression analysis. Moreover, Hickey et al. (2008) have suggested that repeat length variation from 120 to 140 CAG does not substantially alter the Q140 phenotype originally reported by Menalled et al. (2003) for 140 CAG mice. Five 4-month old WT mice, and five 12-month WT mice were also studied. Note that we refer to our mutant mice as Q140, despite the slightly shorter CAG repeat length, to relate our findings to other work on the same mutant strain (in which the first exon of mouse huntingtin was replaced with a human equivalent with ~140 CAG repeats) (Menalled et al., 2003), as others have done as well (Hickey et al., 2008, 2012; Lerner et al., 2012). For histological analysis, mice were deeply anesthetized with 0.2 ml of 35% chloral hydrate in saline, and then exsanguinated by transcardial perfusion with 30 ml of 6% dextran in sodium phosphate buffer (PB), followed by 200 ml of 3.5% paraformaldehyde—0.6% glutaraldehyde—15% saturated picric acid in PB (pH 7.4). The brain of each mouse was removed, postfixed overnight in 3.5% paraformaldehyde—15% saturated picric acid in PB. The right side of the brain was used for a prior light microscopic (LM) (Deng et al., 2013) and the present cortical thickness assessment, and the left for the current electron microscopic (EM) double-label analysis. The left side of the brain had also been used in our prior EM single-label analysis (Deng et al., 2013). For EM studies, forebrain was sectioned at 50 μm on a vibratome.

### EM double-immunolabeling for VGLUT1 or VGLUT2 with dopamine receptor D1

Sections were pretreated with 1% sodium borohydride in 0.1 M PB for 30 min followed by incubation in 0.5% H_2_O_2_solution in 0.1 M PB for 30 min. To carry out conventional double-label immunohistochemistry, sections were incubated overnight at room temperature in primary antisera containing guinea pig anti-VGLUT1 or VGLUT2 (diluted 1:2000) and rat anti D1 (1:400), or rabbit anti-VGLUT2 (diluted 1:2000) and rat anti D1 (1:400) with 0.1 M PB containing 10% normal horse serum, 4% normal goat serum, 1.5% bovine serum albumin, and 0.02% Triton X-100. Sections were then rinsed and incubated in a mixture of biotinylated goat anti-guinea pig IgG diluted 1:100 and goat anti-rat IgG diluted 1:100 (to detect guinea pig anti-VGLUT1 or VGLUT2, and rat anti-D1), or a mixture of goat anti-rabbit IgG 1:100 and goat anti-rat IgG 1:100 (to detect rabbit anti-VGLUT2, and rat anti-D1) in 0.1 M PB (pH 7.4) at room temperature for 1 h. This was followed by incubation in a mixture containing avidin-biotin complex (ABC) and rat peroxidase-antiperoxidase (PAP) at a 1:500 dilution (to detect guinea pig anti-VGLUT1 or VGLUT2, and rat anti-D1), or a mixture of rabbit PAP and rat PAP (to detect rabbit anti-VGLUT2, and rat anti-D1) in 0.1 M PB (pH 7.4) at room temperature for 2 h. The sections were rinsed between secondary and ABC and/or PAP incubations in three 5-min washes of PB. Subsequent to the ABC and/or PAP incubation, the sections were rinsed with three to six 10-min washes in 0.1 M PB, and a peroxidase reaction using diaminobenzidine (DAB) carried out. After the PB rinses, the sections were immersed for 10 min in 0.05% DAB (Sigma, St. Louis, MO) in 0.1 M PB (pH 7.2). Hydrogen peroxide was then added to a final concentration of 0.01%, and the sections were incubated in this solution for an additional 10 min, and washed six times in PB.

### Preparation of tissue for EM

Following immunolabeling as described above, sections processed for EM viewing were rinsed in 0.1 M sodium cacodylate buffer (pH 7.2), postfixed for 1 h in 2% osmium tetroxide (OsO_4_) in 0.1 M sodium cacodylate buffer, dehydrated in a graded series of ethyl alcohols, impregnated with 1% uranyl acetate in 100% alcohol, and flat-embedded in Spurr's resin (Electron Microscopy Sciences, Fort Washington, PA). For the flat-embedding, the sections were mounted on microslides pretreated with liquid releasing factor (Electron Microscopy Sciences, Fort Washington, PA). Pieces of embedded tissue were cut from the dorsolateral (motor) striatum and glued to carrier blocks, and ultrathin sections were cut from these specimens with a Reichert ultramicrotome. The sections were mounted on mesh grids, stained with 0.4% lead citrate and 4.0% uranyl acetate using an LKB Ultrastainer, and finally viewed and images captured with a JEOL 2000EX electron microscope.

### Antibodies

All VGLUT antisera used are highly selective for their target antigens (Fremeau et al., 2001; Montana et al., 2004; Melone et al., 2005; Wässle et al., 2006). The immunogen for the VGLUT1 antibody (Chemicon AB5905) was aa542–560 of the C-terminus of rat VGLUT1, while that for the guinea pig VGLUT2 antibody (Chemicon AB5907) was aa565–582 of the C-terminus of rat VGLUT2. The immunogen for the rabbit VGLUT2 antibody (V2514, Sigma) was aa520–538 near the C-terminus of rat VGLUT2. A previous study of ours demonstrated that the immunolabeling in rat striatum for the two VGLUT2 antibodies used here shows complete colocalization (Lei et al., 2013). The rat anti-D1 antibody (Sigma D-187) is directed against the 97 amino acid C-terminal fragment of human D1 (Hersch et al., 1995; Wang and Pickel, 2002). The antibody is selective for D1 in human, primate and rodent brain (Levey et al., 1993; Hersch et al., 1995; Wang and Pickel, 2002; Lei et al., 2004, 2013).

### EM analysis

**Figure 1:**
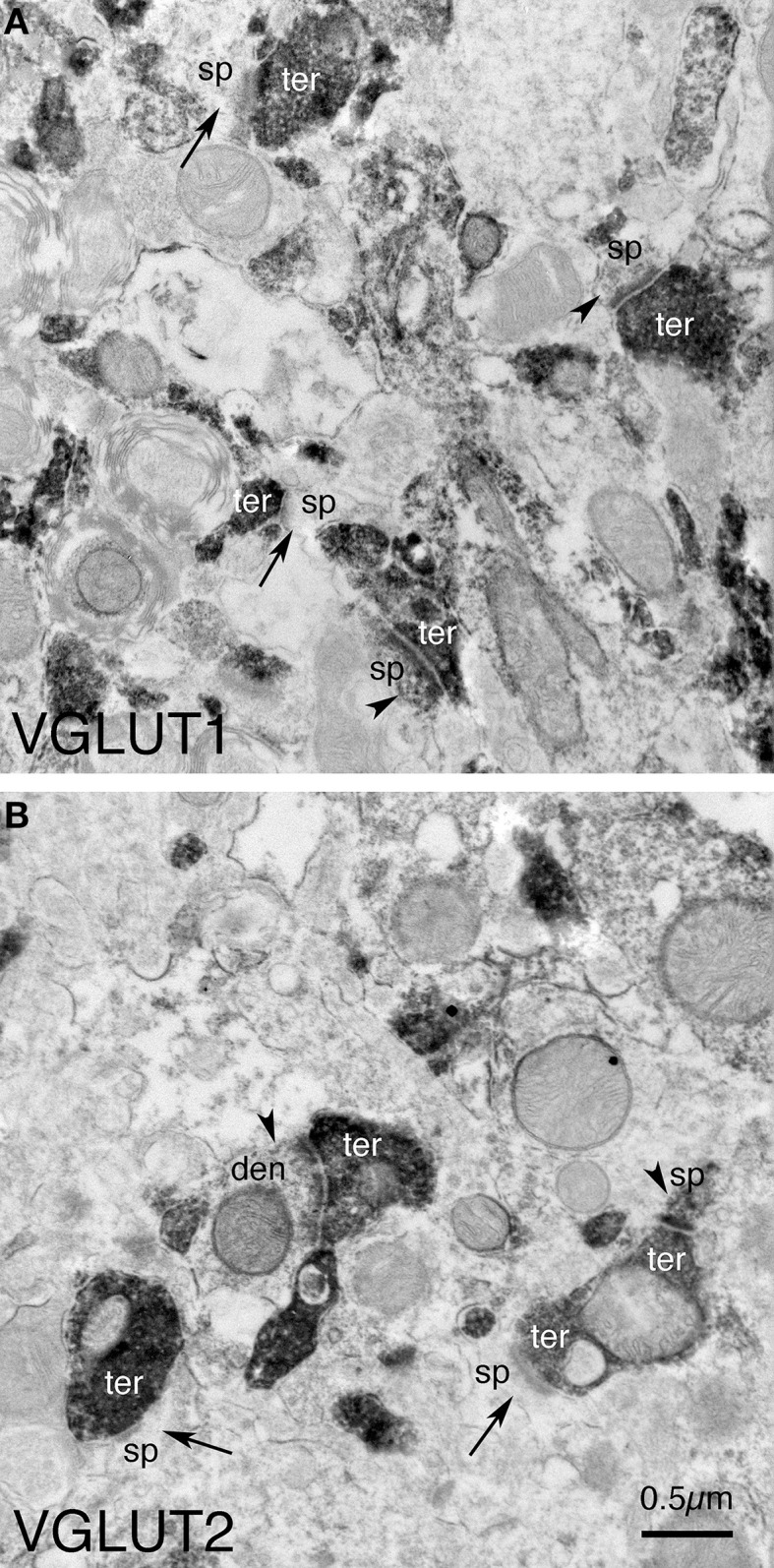
**Examples of the fields of view captured in the EM images used for analysis**. Image **(A)** shows VGLUT1+ immunolabeled synaptic terminals (ter) on D1+ (arrowheads) and D1− (arrows) spines (sp) and dendrites (den) in striatum in WT mice at 12 months of age. Image **(B)** shows VGLUT2+ immunolabeled synaptic terminals (ter) on D1+ (arrowheads) and D1− (arrows) spines (sp) and dendrites (den) in striatum in WT mice at 12 months of age. Both images are at the same magnification.

**Figure 2:**
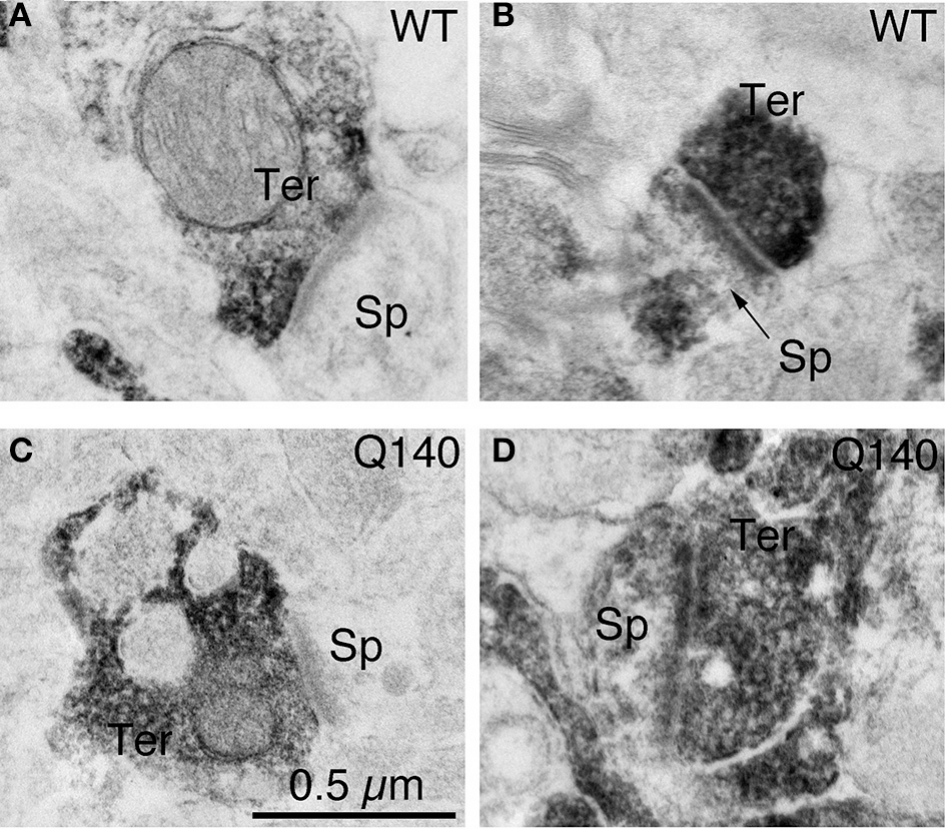
**Examples of EM images of VGLUT1+ immunolabeled synaptic terminals on D1− (A) and D1+ (B) spines in striatum in WT mice at 12 months of age, and of VGLUT1+ immunolabeled synaptic terminals on D1− (C) and D1+ (D) spines in striatum in Q140 mice at 12 months of age**. All images are at the same magnification.

**Figure 3:**
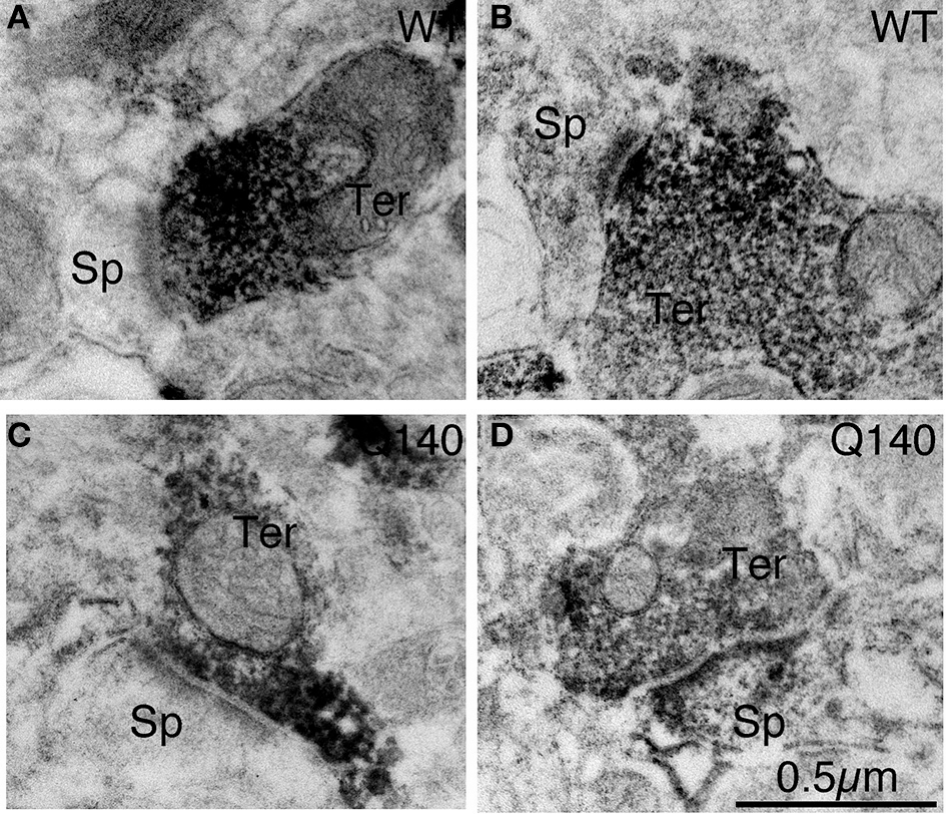
**Examples of EM images of VGLUT2+ immunolabeled synaptic terminals on D1− (A) and D1+ (B) spines in striatum in WT mice at 4 months of age, and of VGLUT2+ immunolabeled synaptic terminals on D1− (C) and D1+ (D) spines in striatum in Q140 mice at 4 months of age**. All images are at the same magnification.

**Figure 4:**
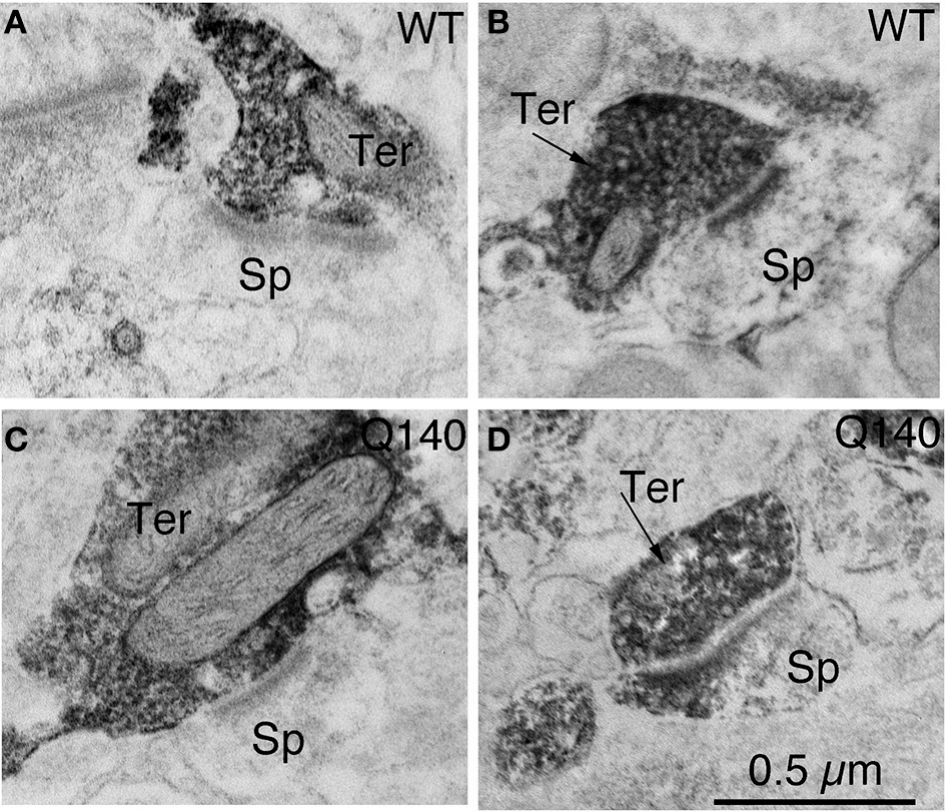
**Examples of EM images of VGLUT2+ immunolabeled synaptic terminals on D1− (A) and D1+ (B) spines in striatum in WT mice at 12 months of age, and of VGLUT2+ immunolabeled synaptic terminals on D1− (C) and D1+ (D) spines in striatum in Q140 mice at 12 months of age**. All images are at the same magnification.

Blinded analysis and quantification was carried out on digital EM images of random fields from dorsolateral somatomotor striatum (Figure 1). Typically, 25–30 EM images that in total encompassed 400–450 μm^2^ of dorsolateral striatum per marker combination were analyzed per animal. This typically yielded 45–55 thalamic and 65–75 cortical terminals per animal per marker combination in WT mice, but fewer in mutant mice, as described in the Results Section. We focused on dorsolateral striatum because matrix compartment neurons of this region are important for motor function, and because it is poor in striosomes (although not entirely devoid), and the major target of intralaminar thalamus (Berendse and Groenewegen, 1990; Gerfen, 1992; Desban et al., 1993; Wang et al., 2007). We performed the analysis on the upper 5 microns of the sections, in which labeling was optimal. We avoided the very surface, where histology was poor. All VGLUT-immunolabeled asymmetric synaptic terminals in each image were tabulated as to their postsynaptic target (spine vs. dendrite, D1+ vs. D1−) and their size measured. We studied eight types of synaptic terminals: (1) VGLUT1 corticostriatal terminals on D1–positive spines of striatal neurons; (2) VGLUT1 corticostriatal terminals on D1–negative spines of striatal neurons; (3) VGLUT2 thalamostriatal terminals on D1–positive spines of striatal neurons; (4) VGLUT2 thalamostriatal terminals on D1–negative spines of striatal neurons; (5) VGLUT1 corticostriatal terminals on D1–positive dendrites of striatal neurons; (6) VGLUT1 corticostriatal terminals on D1–negative dendrites of striatal neurons; (7) VGLUT2 thalamostriatal terminals on D1–positive dendrites of striatal neurons; (8) VGLUT2 thalamostriatal terminals on D1–negative dendrites of striatal neurons. Examples of VGLUT1+ axospinous terminals on D1–positive (D1+) and D1–negative (D1−) spines at 12 months in WT and Q140 mice are shown in Figure 2. Examples of VGLUT2+ axospinous terminals on D1+ and D1− spines in WT and Q140 mice at 4 and 12 months are shown in Figures 3, 4, respectively. Labeled spines and terminals were identifiable by the dark flocculent DAB reaction product, and were typically about twice the darkness of unlabeled structures. Only terminals with an overt synaptic contact possessing a PSD (post-synaptic density) were counted and measured, since all VGLUT terminals are excitatory and their synaptic contacts evidenced by the presence of vesicles in the terminal and a PSD in the target spine or dendrite (Deng et al., 2013; Lei et al., 2013). The size of terminals was determined by measuring them at their widest diameter parallel to and 0.1 μm before the postsynaptic density, and spines were identifiable by their small size, continuity with dendrites, prominent postsynaptic density, and/or the presence of spine apparatus (Wilson et al., 1983). Dendrites were identifiable by their size, oval or elongate shape, and the presence of microtubules and mitochondria. As we have previously noted (Reiner et al., 2010), these measurements were made in random sections that did not necessarily pass through the widest point of each terminal, and thus may underestimate the peak size of the labeled terminals in three dimensions. Nonetheless, we have previously noted based on semi-serial sections that this underestimate is only about 10% (Reiner et al., 2010), and our goal was to compare WT and Q140 in any case, for which any underestimate should be similar. For VGLUT1 and VGLUT2, counts of labeled and unlabeled synaptic terminals on D1–positive or D1–negative spines and dendrites were made to ascertain the percent of axospinous and axodendritic terminals in mouse striatum that possess VGLUT1 or VGLUT2, and to determine the abundance of each terminal type per unit area of striatum. The data are presented as group means (±s.e.m.) for the traits analyzed, unless otherwise stated.

### Open field

Homozygous Q140 mice have been reported to show hypokinesia in open field by 4 months of age, but heterozygous Q140 males (such as those we studied) have not been found to show hypoactivity in open field as late as 9 months of age (Menalled and Howland, personal communication). We thus conducted a 30-min open field test on 12 month-old Q140 and WT mice, but not on 4-month old mice, using a Noldus EthoVision video tracking system (Noldus Information Tecknology, Netherlands), and the SEE software of Drai and Golani (2001). Our goal was to determine if there are motor deficits in our heterozygous Q140 males at 12 months of age, and if so relate them to the defects in cortical and thalamic input to SPNs. The circular open field arena has a 200 cm diameter, a non-porous gray floor, and a 50 cm gray wall, which provides contrast for video tracking of mice. SEE dichotomizes mouse movements into lingering episodes and progression movements, and provides endpoints related to locomotion that are robust in identifying differences among mouse strains (Drai et al., 2000; Drai and Golani, 2001; Kafkafi et al., 2001, 2003; Lipkind et al., 2004), and between R6/2 HD and WT mice (Reiner et al., 2012a).

### Cortical thickness

As part of an effort to determine if cortical pathology was present, we measured the thickness of primary motor cortex (M1) in our 1-year old mice, since it is among the first affected cortical areas in human HD that shows thinning (Rosas et al., 2005), and a major source of input to rodent dorsolateral motor striatum (Reiner et al., 2003, 2010). Thickness in the present study was measured in a series of cresyl violet-stained sections from the right hemisphere, from Bregma level 1.94 to Bregma level −0.94. These sections had been prepared as part of Deng et al. (2013). Blinded measurements were made of the depth of M1 cortex perpendicular to the cortical surface at the midpoint of M1 in each section, using Image J software. Typically seven sections were measured per animal, and mean thickness determined for each case from these measurements.

### Statistical analyses

Because of the sample size, nonparametric statistics were used to statistically evaluate differences in terminal size and spatial abundance, within and between genotypes, for D1+ vs. D1− targets. For comparisons within genotype for VGLUT+ terminals on D1+ vs. D1− targets, the Wilcoxon signed-rank test was used. For comparisons between genotypes for VGLUT+ terminals on either D1+ or D1− targets, the Mann-Whitney test was used. In the case of the VGLUT terminal size frequency distribution data, the differences between WT and Q140 mice for a given terminal type were analyzed using repeated measures ANOVA. Linear regression was used to assess the relationship between four distinct open field motor endpoints and the abundance of corticostriatal or thalamostriatal synaptic terminals on dSPN or iSPN spines in striatum of 12 month-old Q140 and WT mice. The significance level was set at *p* ≤ 0.01 to adjust for multiple comparisons that were performed in the case of the various EM and behavioral data sets. The significance level was *p* ≤ 0.05 in the case of the *t*-test used to assess the cortical thickness difference between WT and Q140 mice.

## Results

### EM double-immunolabeling studies

VGLUT1 corticostriatal terminals on D1+ or D1− dendrites of striatal neurons are the least common of the terminal types examined (about 7% of all corticostriatal terminals), and we thus collected only a small number of instances of them on either target in WT or Q140 mice. Though they showed a trend toward decline in abundance at 12 months in Q140 mice, the results were variable due to the scarcity of axodendritic corticostriatal terminals in mice. Thus, we do not present data on VGLUT1 corticostriatal terminals on D1+ vs. D1− striatal dendrites here. Results for the other corticostriatal or thalamostriatal terminal types are presented below.

#### VGLUT1 axospinous terminals

**Figure 5:**
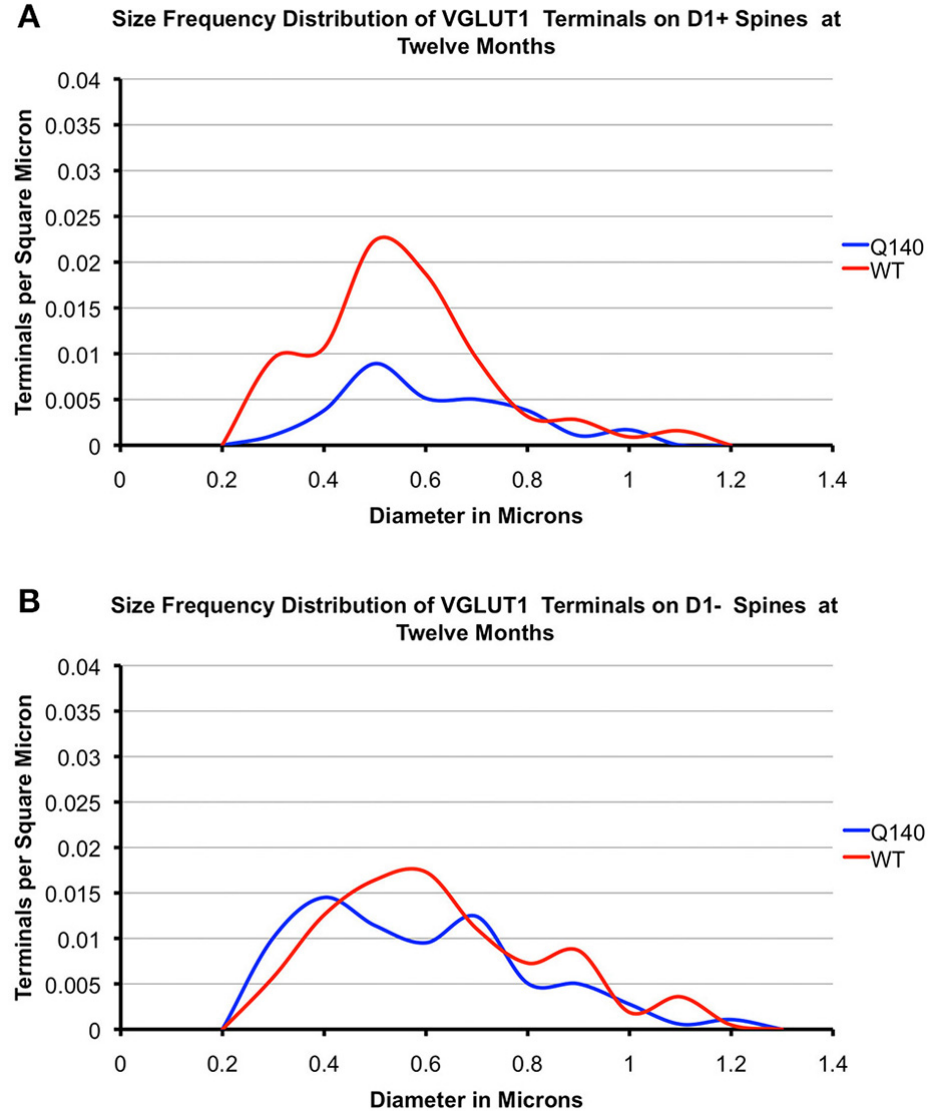
**Graphs showing the size frequency distributions for VGLUT1+ axospinous synaptic terminals on D1+ (A) and D1− (B) striatal projection neurons in striatum of 12 month-old WT and heterozygous Q140 mice**. Note that the large shortfall in small terminals on D1+ spines in Q140 mice.

As shown in Table 1, the mean spatial abundance of VGLUT1+ axospinous terminals on D1+ spines was similar to that of VGLUT1+ axospinous terminals on D1− spines (Table 1), as also noted in Doig et al. (2010). The mean size of VGLUT1-positive (VGLUT1+) axospinous terminals on D1+ spines was, however, less than that of VGLUT1+ axospinous terminals on D1− spines in WT mice, and the difference trended toward statistical significance (*p* = 0.0625). Consistent with this, the size frequency distributions show that large terminals (> 0.7 μm) were more common on D1− than D1+ spines in WT mice (Figure 5). The results for Q140 mice were very different than for WT mice. In particular, the spatial abundance of VGLUT1+ synaptic terminals on D1+ spines was strikingly and significantly reduced (by 63.3%) in Q140 mice at 12 months, compared to 12-month old WT mice (*p* = 0.0079). Moreover, unlike in WT mice, the mean size of VGLUT1+ axospinous terminals on D1+ spines in Q140 mice at 12 months was not less than that of VGLUT1+ axospinous terminals on D1− spines. In fact, it was greater, but not significantly so. The size frequency distribution of the VGLUT1+ axospinous terminals on D1+ spines for WT and Q140 mice shed further light on these differences. Overall, the size frequency distribution of VGLUT1+ axospinous terminals on D1+ spines for Q140 mice was significantly different (*p* = 0.0001) from that for WT mice (Figure 5). The size frequency distribution graphs also revealed that the D1+ spines in Q140 mice showed a particular depletion of smaller terminals (i.e., < 0.6 μm), thus explaining the trend toward a larger mean size of VGLUT1+ axospinous terminals on D1+ spines in Q140 mice than WT mice. In contrast to D1+ spines, the mean abundance of VGLUT1+ terminals on D1− spines was not significantly different between Q140 and WT mice (*p* = 0.2222). Consistent with this, the size frequency distribution of the VGLUT1+ axospinous terminals on D1− spines in Q140 mice was not significantly different from that for VGLUT1+ axospinous terminals on D1− spines in WT mice (*p* = 0.1975). Thus, the loss of axospinous VGLUT1+ corticostriatal terminals in Q140 mice at 12 months is highly preferential for D1+ spines, and seems to especially involve smaller terminals. Note that our prior study indicates that the decline in spatial abundance of VGLUT1+ axospinous terminals in Q140 mice at 12 months did not stem from a failure to label otherwise surviving corticostriatal terminals, but rather appears to reflect true terminal loss. VGLUT1-negative axospinous terminals were observed in the same frequency as VGLUT2+ axospinous terminals in WT and Q140 mice, meaning there was not a disproportionate increase in VGLUT1-unlabeled corticostriatal terminals in Q140 mice (Deng et al., 2013).

**Table 1:** **Abundance and size of VGLUT1-positive axospinous terminals in dorsolateral striatum in 12-month old WT and Q140 mice**.

**Measured terminal trait**	**WT D1+ spine**	**WT D1− spines**	**Q140 D1+ spine**	**Q140 D1− spines**
Terminal abundance (per μm^2^)	0.082 ± 0.006	0.084 ± 0.006	0.030 ± 0.001^*^	0.074 ± 0.005
Terminal abundance (as % WT)	100.0%	100.0%	36.7%^*^	88.0%
Axospinous terminal size (μm)	0.602 ± 0.014	0.676 ± 0.013	0.667 ± 0.034	0.627 ± 0.008
Axospinous terminal size (as % WT)	100.0%	100.0%	110.8%	92.7%

* *p = 0.0079–Q140 D1+ axospinous terminal abundance compared to WT D1+ axospinous terminal abundance*.

#### VGLUT2 axospinous terminals

**Figure 6:**
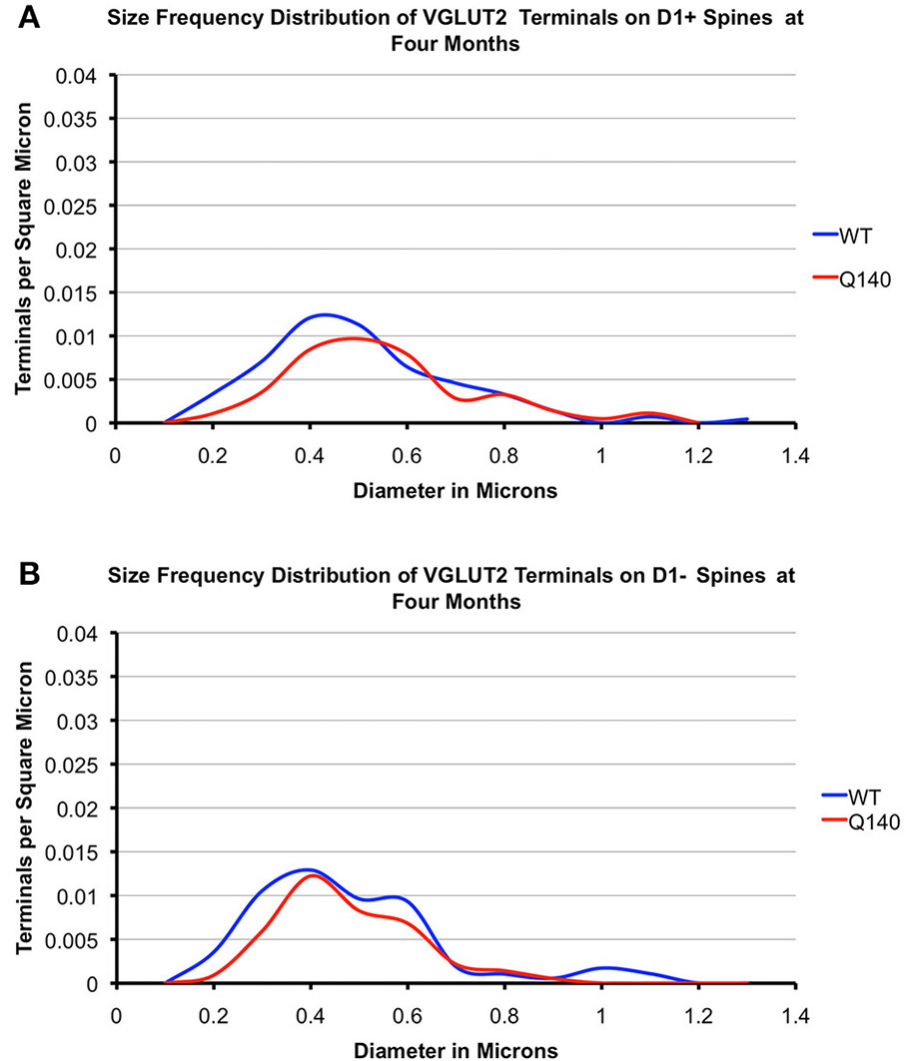
**Graphs showing the size frequency distributions for VGLUT2+ axospinous synaptic terminals on D1+ (A) and D1− (B) striatal projection neurons in striatum of 4 month-old WT and heterozygous Q140 mice**. Note that the shortfall in VGUT2+ axospinous terminals on both D1+ and D1− spines in Q140 mice.

**Figure 7:**
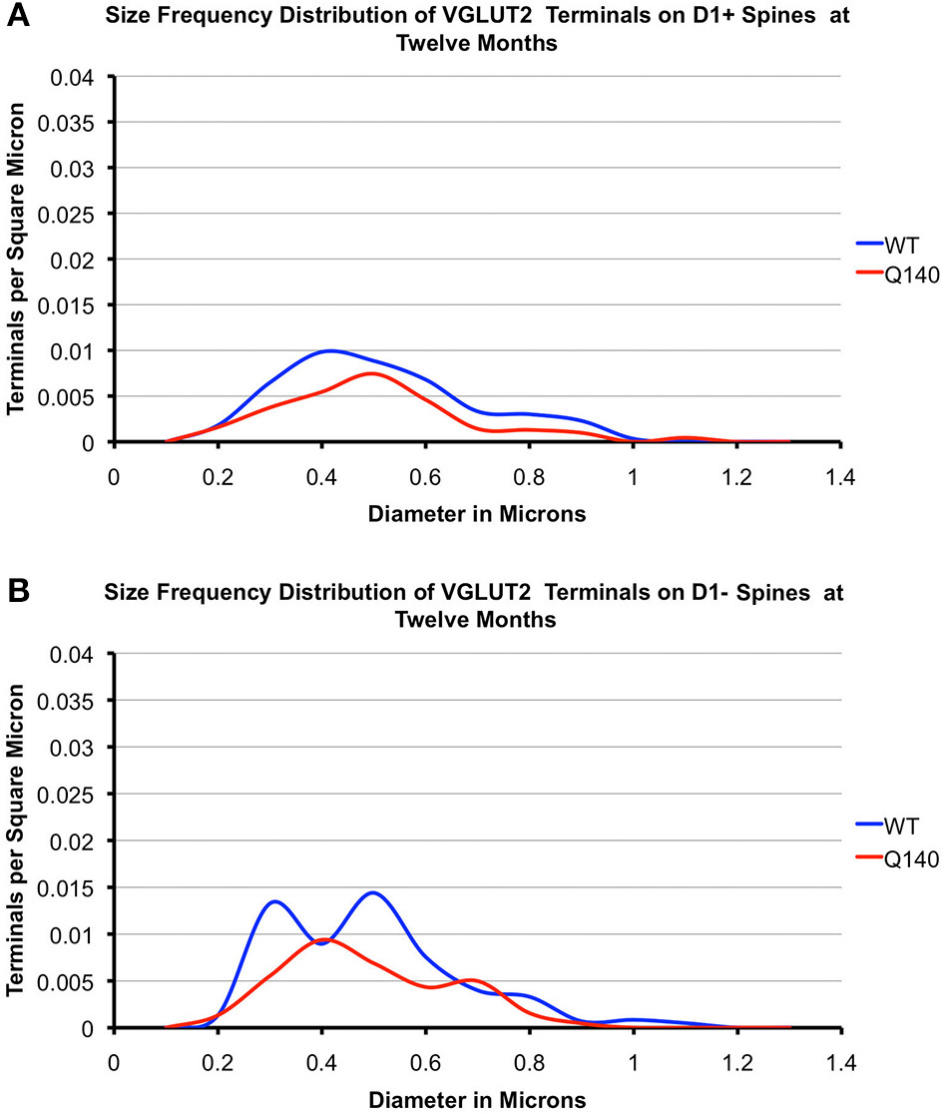
**Graphs showing the size frequency distributions for VGLUT2+ axospinous synaptic terminals on D1+ (A) and D1− (B) striatal projection neurons in striatum of 12 month-old WT and heterozygous Q140 mice**. Note that the shortfall in VGUT2+ axospinous terminals on both D1+ and D1− spines in Q140 mice.

As shown in Tables 2, 3, the mean spatial abundance of VGLUT2+ terminals on D1+ spines was similar to that for D1− spines in WT mice at both 4 and 12 months. The mean size of VGLUT2+ terminals on D1+ spines in WT mice was also not significantly different from that of VGLUT2+ terminals on D1− spines in WT mice, at either 4 or 12 months. The size frequency distribution data did, however, reveal that VGLUT2+ axospinous thalamostriatal terminals on D1+ spines in WT mice had a unimodal distribution, with a peak at 0.4 μm (Figures 6, 7). By contrast, VGLUT2 axospinous thalamostriatal terminals on D1− spines in WT mice showed a bimodal distribution (more notably at 12 months), with peaks at 0.3–0.4 μm and 0.5–0.6 μm. In the case of the Q140 mutant mice, the mean abundance of VGLUT2+ axospinous synaptic terminals on both D1+ and D1− spines was reduced by 30–40% compared to WT mice at both 4 and 12 months. The difference was significant for D1+ spines at both ages (4-months: *p* = 0.0079; 12-months: *p* = 0.0079), and at 12 months for D1− spines (*p* = 0.0079). Mean VGLUT2 axospinous terminal size was, however, no different between Q140 and WT mice for either VGLUT2 terminals on D1+ spines or those on D1− spines. Similar overall effects were seen in the size frequency distributions (Figures 6, 7). Significant differences were seen between Q140 and WT for VGLUT2+ axospinous terminals on D1+ spines at both 4 months (*p* = 0.0028) and 12 months (*p* = 0.0020), but a significant difference for D1− spines was only seen at 12 months (*p* = 0.0019). Thus, the loss of VGLUT2+ terminals from D1+ spines was not progressive between 4 and 12 months, but that for D1− spines appeared to be perhaps progressive. For both VGLUT2+ terminals on D1+ and on D1− spines in Q140 mice, the loss occurred at both the higher and lower ends of the size ranges, explaining why the mean size of VGLUT2+ axospinous terminals in Q140 mice was unaltered compared to WT mice. Note again that our prior study (Deng et al., 2013) indicated that the shortfall in the spatial abundance of VGLUT2+ axospinous terminals in Q140 mice did not stem from a failure to label otherwise surviving thalamostriatal terminals, but rather appears to reflect true terminal shortfall. VGLUT2-negative axospinous terminals were observed in the same frequency as VGLUT1+ axospinous terminals in WT and Q140 mice, meaning there was not a disproportionate increase in VGLUT2-unlabeled thalamostriatal terminals in Q140 mice.

**Table 2:** **Abundance and size of VGLUT2-positive axospinous terminals in dorsolateral striatum of 4-month old WT and Q140 mice**.

**Measured terminal trait**	**WT D1+ spine**	**WT D1− spines**	**Q140 D1+ spine**	**Q140 D1− spines**
Terminal abundance (per μm^2^)	0.051 ± 0.002	0.052 ± 0.003	0.037 ± 0.002^*^	0.033 ± 0.004
Terminal abundance (as % WT)	100.0%	100.0%	72.1%^*^	64.1%
Axospinous terminal size (μm)	0.551 ± 0.017	0.526 ± 0.007	0.594 ± 0.022	0.528 ± 0.020
Axospinous terminal size (as % WT)	100.0%	100.0%	107.9%	100.5%

* *p = 0.0079–Q140 D1+ axospinous terminal abundance compared to WT D1+ axospinous terminal abundance*.

**Table 3:** **Abundance and size of VGLUT2-positive axospinous terminals in dorsolateral striatum of 12-month old WT and Q140 knock-in mice**.

**Measured terminal trait**	**WT D1+ spine**	**WT D1− spines**	**Q140 D1+ spine**	**Q140 D1− spines**
Terminal abundance (per μm^2^)	0.044 ± 0.003	0.054 ± 0.003	0.027 ± 0.001^*^	0.035 ± 0.002^**^
Terminal abundance (as % WT)	100.0%	100.0%	61.9%^*^	63.6%^**^
Axospinous terminal size (μm)	0.560 ± 0.023	0.542 ± 0.014	0.556 ± 0.024	0.533 ± 0.013
Axospinous terminal size (as % WT)	100.0%	100.0%	99.2%	98.3%

* *p = 0.0079–Q140 D1+ axospinous terminal abundance compared to WT D1+ axospinous terminal abundance*.

** *p = 0.0079–Q140 D1− axospinous terminal abundance compared to WT D1− axospinous terminal abundance*.

#### VGLUT2 axodendritic terminals

**Figure 8:**
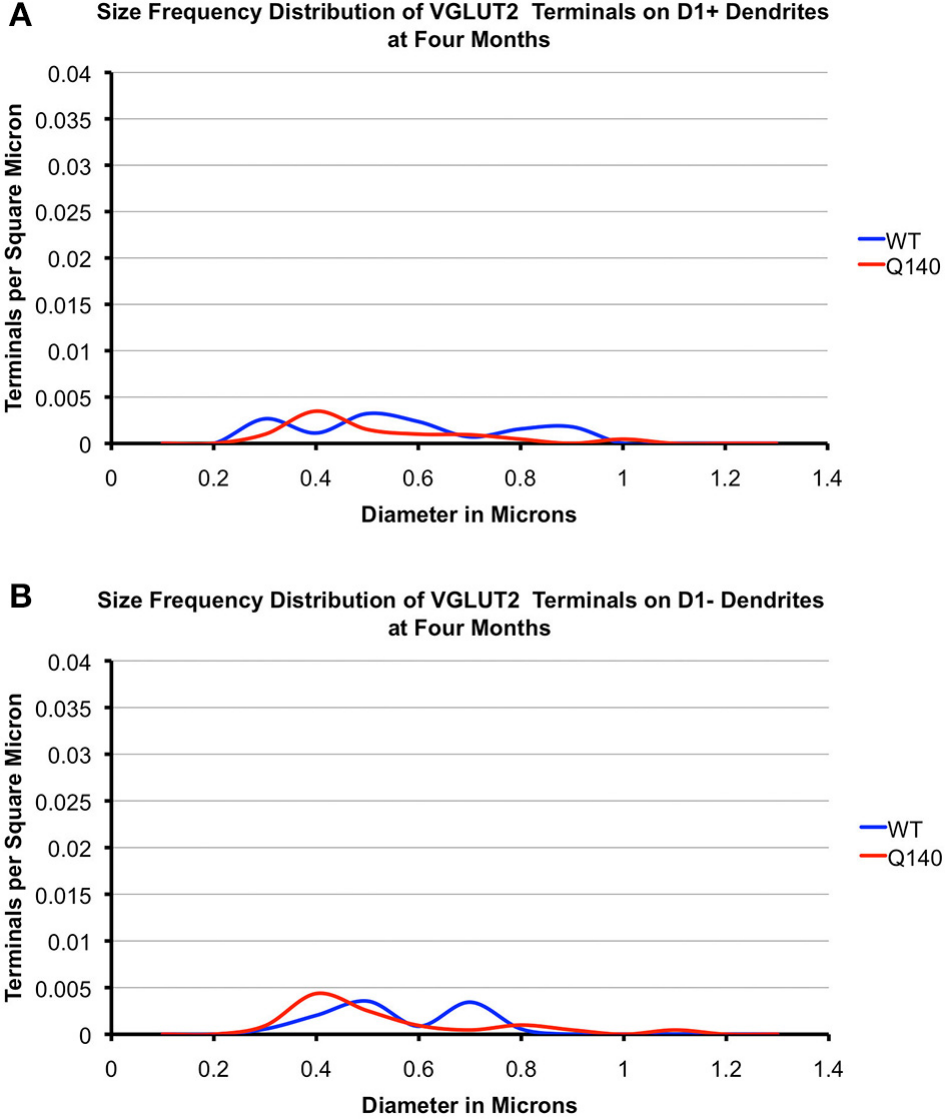
**Graphs showing the size frequency distributions for VGLUT2+ axodendritic synaptic terminals on D1+ (A) and D1− (B) striatal projection neurons in striatum of 4 month-old WT and heterozygous Q140 mice**. Note that VGUT2+ axodendritic terminals on both D1+ and D1− spines are largely similar in abundance in WT and Q140 mice, but with some possible shortfall in large terminals on D1− dendrites.

**Figure 9:**
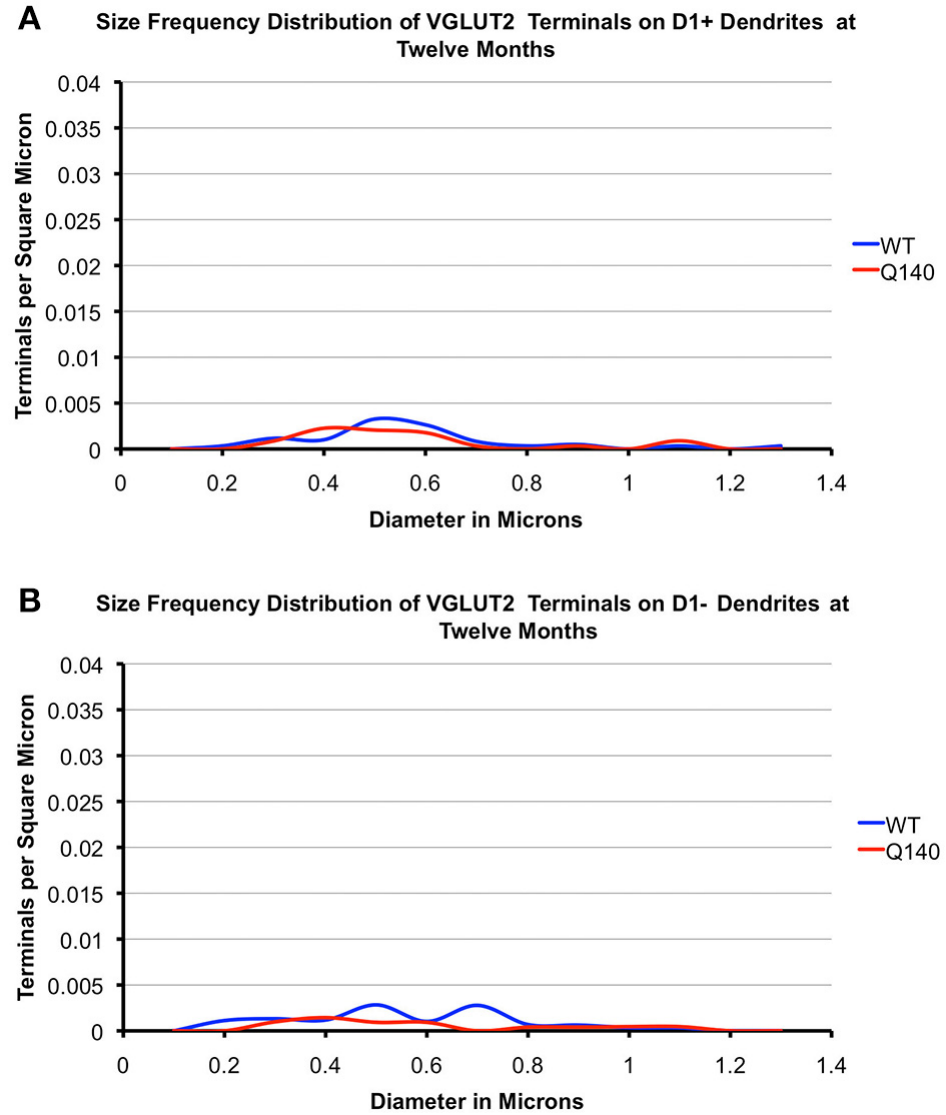
**Graphs showing the size frequency distributions for VGLUT2+ axodendritic synaptic terminals on D1+ (A) and D1− (B) striatal projection neurons in striatum of 12 month-old WT and heterozygous Q140 mice**. Note that VGUT2+ axodendritic terminals on both D1+ and D1− spines are largely similar in abundance in WT and Q140 mice, but with some possible shortfall in large terminals on D1− dendrites.

As shown in Tables 4, 5, the mean spatial abundance of VGLUT2+ synaptic terminals on D1+ dendrites was indistinguishable from that on D1− dendrites for WT mice, at both 4 and 12 months of age. The mean size of VGLUT2+ synaptic terminals on D1+ dendrites was also indistinguishable from that on D1− dendrites for WT mice, at both ages. Overall, VGLUT2+ axodendritic endings in WT mouse striatum were far less common than VGLUT2+ axospinous endings, with the axospinous to axodendritic ratio for VGLUT2 synaptic terminals being about 4–1. The Q140 mice showed no consistent or significant differences in the mean spatial abundance of VGLUT2 axodendritic terminals on D1+ vs. D1− dendrites at either 4 or 12 months. They also showed no significant differences from WT in mean spatial abundance for either axodendritic terminal type at either age. The mean size of VGLUT2+ synaptic terminals on D1+ dendrites in Q140 mice was also indistinguishable from that on D1− dendrites for both age groups, and the mean size of VGLUT2+ synaptic terminals on dendrites of either type was also indistinguishable at either age from that in WT mice. The size frequency distributions (Figures 8, 9), however, suggested a possible loss of larger VGLUT2 axodendritic thalamostriatal terminals on D1− spines in Q140 mice at both ages. Statistical analysis, however, did not detect significant differences between WT and Q140 in the size frequency distributions for VGLUT2+ terminals on D1+ or D1− dendrites at either age. Our overall results suggest loss of axodendritic VGLUT2+ terminals in Q140 mice was not prominent, but more detailed study will be needed to determine if there is slight loss of larger VGLUT2+ synaptic terminals from D1− dendrites.

**Table 4:** **Abundance and size of VGLUT2-positive axodendritic terminals in dorsolateral striatum of 4-month old WT and Q140 knock-in mice**.

**Measured terminal trait**	**WT D1+ dendrite**	**WT D1− dendrite**	**Q140 D1+ dendrite**	**Q140 D1− dendrite**
Terminal abundance (per μm^2^)	0.013 ± 0.002	0.011 ± 0.003	0.009 ± 0.003	0.011 ± 0.002
Terminal abundance (as % WT)	100.0%	100.0%	65.9%	101.0%
Axodendritic terminal size (μm)	0.583 ± 0.060	0.607 ± 0.046	0.581 ± 0.035	0.569 ± 0.048
Axodendritic terminal size (as % WT)	100.0%	100.0%	99.7%	93.6%

**Table 5:** **Abundance and size of VGLUT2-positive axodendritic terminals in dorsolateral striatum of 12-month old WT and Q140 knock-in mice**.

**Measured terminal trait**	**WT D1+ dendrites**	**WT D1− dendrites**	**Q140 D1+ dendrites**	**Q140 D1− dendrites**
Terminal abundance (per μm^2^)	0.010 ± 0.002	0.013 ± 0.002	0.009 ± 0.001	0.006 ± 0.002
Terminal abundance (as % WT)	100.0%	100.0%	88.1%	41.6%
Axodendritic terminal size (μm)	0.610 ± 0.028	0.622 ± 0.026	0.601 ± 0.040	0.646 ± 0.078
Axodendritic Terminal size (as % WT)	100.0%	100.0%	98.6%	103.8%

### Correlation between open field motor endpoints and VGLUT terminal loss

Q140 mice showed a mild hypokinesia at 12 months, which was reflected in several open field motor parameters. For example, Q140 mice showed a decrease in overall movement, progression segment length (*p* = 0.003), and maximum speed, and an increase in the number of pauses (*p* = 0.002) (Table 6). Of note, distance traveled, progression segment length and maximum speed were directly correlated with the abundance of VGLUT1+ corticostriatal terminals on D1+ spines across the WT and Q140 mice (i.e., *n* = 10), while number of pauses was inversely correlated. The correlations for progression segment length and stops were highly significant. By contrast, none of these motor parameters was significantly correlated with the abundance of VGLUT1+ axospinous terminals on D1− spines. Similarly, VGLUT2+ axospinous thalamostriatal terminal abundance on neither D1+ nor D1− spines was significantly correlated with any of these motor endpoints. Thus, the loss of VGLUT1+ axospinous terminals on D1+ spines at 12 months is significantly and selectively linked to the mild hypokinesia seen in the Q140 mice.

**Table 6:** **Correlation between open field behavior and VGLUT terminal loss in 1-year old Q140 and WT mice**.

	**Distance traveled in centimeters**	**Progression segment length in centimeters**	**Maximum speed in cm/sec**	**Number of stops per unit distance**
Q140 as % WT	84.2% (*p* = 0.085)	**76.4% (*p* = 0.003)**	89.3% (*p* = 0.074)	**130.3% (*p* = 0.002)**
Correlation with VG1+ terminals on D1+ spines	0.653 (*p* = 0.0366)	**0.900 (*p* = 0.0003)**	0.680 (*p* = 0.0292)	**−0.846 (*p* = 0.0014)**
Correlation with VG1+ terminals on D1− spines	0.335 (*p* = 0.344)	0.485 (*p* = 0.155)	0.441 (*p* = 0.202)	−0.570 (*p* = 0.060)
Correlation with VG2+ terminals on D1+ spines	0.304 (*p* = 0.394)	0.629 (*p* = 0.052)	0.271 (*p* = 0.448)	−0.716 (*p* = 0.0229)
Correlation with VG2+ terminals on D1− spines	0.187 (*p* = 0.606)	0.579 (*p* = 0.080)	0.251 (*p* = 0.485)	−0.598 (*p* = 0.086)

*With p < 0.01 required for significance (Bold)*.

### Cortical thickness

The thickness of M1 in Q140 mice at 12 months was 1.30 ± 0.03 mm, compared to 1.33 ± 0.03 mm in WT mice at 12 months. This difference was not significant by a *t*-test (*p* = 0.405).

## Discussion

In the present study, we found that dSPNs show a substantial and selective loss of about 65% of axospinous cortical input in Q140 mice by 12 months of age (Deng et al., 2013). Axospinous cortical input to iSPNs was, however, largely unchanged. In our prior study, we also reported that striatal volume in 12-month old Q140 mice was unchanged. In the present study, we also found no evidence of cortical thinning in Q140 mice at 12 months of age. Additionally, Rising et al. (2011) did not observe cortical neuron loss in Q140 mice at this age. Thus, the preferential loss of cortical terminals on D1+ spines is unlikely to be due to either cortical or striatal neuron loss *per se*, but rather selective loss of axospinous terminals from dSPN spines during disease progression. By contrast to the corticostriatal projection, loss of axospinous thalamic input to dSPNs and iSPNs in Q140 mice was comparable, and already evident at 4 months of age. The loss of cortical input to D1+ spines at 12 months of age was highly correlated with a slight but significant decrease in locomotor activity in open field, but loss of thalamic input was not. The implications of our findings for the pathophysiology and pathogenesis of human HD are discussed in more detail below.

### Cortical input loss to striatal projection neurons in HD

Numerous imaging studies have reported cortical thinning in premanifest HD, coupled with cortical white matter loss (DiProspero et al., 2004; Kipps et al., 2005; Reading et al., 2005; Rosas et al., 2005, 2006; Ciarmiello et al., 2006; Paulsen et al., 2006; Hobbs et al., 2010a; Aylward et al., 2011; Dumas et al., 2012), and striatal hypoactivation (Grafton et al., 1992; Paulsen et al., 2004; Ciarmiello et al., 2006; Wolf et al., 2012). Although premanifest cortical and striatal neuron loss have not been quantified, they are generally thought to be minimal (Vonsattel et al., 1985; Augood et al., 1997; Vonsattel and DiFiglia, 1998; Glass et al., 2000; Deng et al., 2004; Nopoulos et al., 2010). In this context then, our results in Q140 mice are of interest, as they suggest that corticostriatal synaptic pruning may occur during premanifest HD preferentially on direct pathway neurons. Such premanifest corticostriatal terminal loss might be expected as an early reflection of a pathogenic process that in symptomatic HD causes significant regional thinning of cerebral cortex and loss of cortical pyramidal neurons (Rosas et al., 2003; Kassubek et al., 2004b; Douaud et al., 2006; Mühlau et al., 2007).

Although loss of corticostriatal input prior to significant striatal neuron loss has not been directly demonstrated neuropathologically in either human HD or in prior studies of mouse HD models, other types of data from mouse models are consistent with our findings in Q140 mice. For example, loss of presynaptic markers such as Lin7b and synaptophysin from cortex, loss of postsynaptic markers such as PSD-95 from striatum, loss of dendritic spines from SPNs, and/or loss of excitatory synaptic terminals in striatum are observed in early symptomatic R6/2 and YAC128 mice (Klapstein et al., 2001; Cepeda et al., 2003; Graham et al., 2009; Cummings et al., 2010; Singaraja et al., 2011). Data from R6/2 and YAC128 HD mice suggest that dSPNs in particular show reduced glutamatergic corticostriatal excitation at ages at which there is no loss of dSPNs (Benn et al., 2007; André et al., 2011a,b; Reiner et al., 2012a,b). The small size of the corticostriatal terminals preferentially lost in Q140 mice from dSPNs by 12 months of age in our study suggests that they may predominantly represent input from intratelencephalically projecting (IT)—type corticostriatal neurons (Wilson, 1987; Wright et al., 1999, 2001; Reiner et al., 2003, 2010), which our work and that of others suggests represent the main but not exclusive source of cortical input to the spines of dSPNs (Lei et al., 2004; Cepeda et al., 2008; Reiner et al., 2010; Spigolon et al., 2013; Wall et al., 2013). Such loss of drive to the “go” neurons of the direct pathway would be expected to cause behavioral hypoactivity (Albin et al., 1989; Kravitz et al., 2010; Spigolon et al., 2013), which is observed as a major symptom as both heterozygous and homozygous Q140 mice age (Menalled et al., 2003; Hickey et al., 2008; Rising et al., 2011). Loss of cortical drive to dSPNs may thus explain the significant correlation we found specifically between the abundance of axospinous cortical terminals and motor activity in open field across 12-month old WT and heterozygous Q140 mice. If a similar event occurs in humans, it could help explain the growing motor slowing evident in premanifest HD (Siemers et al., 1996; de Boo et al., 1997; Kirkwood et al., 1999, 2000; Blekher et al., 2004; Rao et al., 2008; Biglan et al., 2009; Bechtel et al., 2010; Delval et al., 2011; Rao et al., 2011; Tabrizi et al., 2011; Turner et al., 2011). It is possible, however, that the link we observed between loss of axospinous terminals from dSPN spines and motor slowing in 12-month Q140 mice reflects the common action of a general disease-related decline. Nonetheless, we saw no correlation between thalamic terminal loss to either dSPN or iSPN spines on one hand and motor slowing on the other. Such a correlation might be expected if terminal loss from SPNs and motor slowing represent the common deleterious action of a generalized disease-related decline on brain pathology and motor abnormalities.

In both R6/2 and YAC128 HD mice, the loss in corticostriatal drive to the striatum is preceded by earlier corticostriatal hyperactivity (Cepeda et al., 2003; Rebec et al., 2006; Joshi et al., 2009; André et al., 2011a). Corticostriatal terminal dysfunction, changes in membrane properties of SPNs, and altered potassium uptake by astroglia all appear to contribute to the SPN hyperactivity (Cepeda et al., 2003; André et al., 2011a; Tong et al., 2014). The direct pathway neurons in particular show early enhanced and later reduced corticostriatal excitation (André et al., 2011a,b). The reduced corticostriatal excitation reflects the loss of corticostriatal input rather than reduced striatal excitability, since dSPNs remain more depolarized at rest, and have elevated input resistances (Cepeda et al., 2003; Singaraja et al., 2011; Estrada-Sanchez and Rebec, 2013). The preferential loss of cortical input to dSPNs is of interest in light of the possibility that the loss is neuroprotective. The dSPNs projecting to the internal pallidal segment (GPi) are the most resistant projection neuron type in HD (Deng et al., 2004), and a downregulation in excitatory cortical input to them could help explain not only why striato-GPi dSPNs resist death better in human HD than do other striatal SPNs, but also may explain the emergence of resistance to corticostriatal excitotoxicity as HD mice age (Hansson et al., 1999, 2001; Graham et al., 2009). The corticostriatal synaptic pruning may, thus, involve activity-dependent mechanisms, rather than an HD-driven cortical pathology selective for the cortical input to dSPNs (Tian et al., 2010; Schafer et al., 2012). To this end, it would also be useful to know if HD differentially affects the two types of corticostriatal neurons, the IT-type and the pyramidal tract-type (or PT-type) (Reiner et al., 2010), and their synapse formation with their striatal target neurons. For example, it could be the case that the HD mutation more greatly affects the behavior of IT-type neurons, rendering them more active than PT-type neurons, which ultimately then leads to the preferential loss of IT-type terminals from dSPN spines.

Our finding of loss of axospinous cortical input to dSPNs but not to iSPNs at 12 months of age in Q140 heterozygous mice is relevant to the possible role of brain-derived neurotrophic factor (BDNF) insufficiency in HD pathogenesis. A number of studies have shown that striatal neurons depend on BDNF for survival (Mizuno et al., 1994; Widmer and Hefti, 1994; Nakao et al., 1995; Martınez-Serrano and Bjorklund, 1996; Alcantara et al., 1997; Ivkovic and Ehrlich, 1999; Aggerman and Ernfors, 2003; Grosse et al., 2005; Ventimiglia et al., 1995), and production and delivery of BDNF from cortex to striatum is diminished in HD and animal models of HD (Cattaneo et al., 2001, 2005; Zuccato et al., 2001, 2003, 2005, 2008; Gauthier et al., 2004; Zuccato and Cattaneo, 2007; Reiner et al., 2012b). Moreover, studies in various mutant mice indicate that diminished cortical production of BDNF harms striatal neurons (Gorski et al., 2003; Baquet et al., 2004; Canals et al., 2004; Saylor et al., 2006; Strand et al., 2007), and intrastriatal BDNF delivery or selective forebrain overexpression of BDNF improves symptoms in transgenic HD mice (Canals et al., 2004; Gharami et al., 2008; Xie et al., 2010; Giralt et al., 2011). Under these circumstances, preferential loss of axospinous cortical terminals from dSPNs might be expected to cause their greater vulnerability in HD and HD models than iSPNs. As has been shown, nonetheless, iSPNs are more vulnerable in both human HD and genetic models of HD (Reiner et al., 1988; Glass et al., 2000; Menalled et al., 2000; Sun et al., 2002; Canals et al., 2004; Deng et al., 2004). A number of lines of evidence, however, show that iSPNs are much more vulnerable than dSPNs to BDNF deprivation (Canals et al., 2004; Baydyuk et al., 2011; Reiner et al., 2012b). Moreover, BDNF production by the type of neuron whose axospinous input to dSPNs is lost (i.e., the IT-type) appears to be less than for the other major type of corticostriatal neuron, the PT-type (Doyle et al., 2008), which is the preferential source of axospinous input to iSPNs (Reiner et al., 2010). Thus, if IT-type terminals are preferentially lost from dSPNs early in premanifest human HD gene carriers, the lesser dependence of dSPNs on BDNF and the lesser BDNF production by IT-type neurons may explain why this does not cause the dSPNs to be the more vulnerable neuron type in HD.

Although the loss of cortical input to dSPNs may help explain the hypokinesia seen very early in the course of HD before striatal neuron loss, as in our 12-month old Q140 heterozygous mice, hyperactivity in a rearing test has been reported in homozygous Q140 mice at 1 month of age (Menalled et al., 2003). Hyperactivity in a rearing test has not, however, been observed at 2.5 months of age in Q140 heterozygous mice, in whom the phenotype is slowed compared to Q140 homozygous mice (Rising et al., 2011). Thus, it is uncertain that Q140 heterozygous mice show an early hyperactivity similar to that reported in Q140 homozygous mice. In any event, the basis of the rearing hyperactivity in homozygous Q140 mice at 2 months of age is uncertain, and not likely to be attributable to cortical input loss since the loss we observed here in heterozygous Q140 mice does not occur until much later. In our prior EM single-label study of Q140 heterozygous mice (Deng et al., 2013), we found a deficiency in thalamic input to striatal dendrites already at 1 month of age, which persists beyond this age. As thalamic input ends on the dendritic shafts of both cholinergic interneurons and striatal projection neurons (Lapper and Bolam, 1992; Sidibé and Smith, 1999; Salin and Kachidian, 1998; Giorgi et al., 2001; Bacci et al., 2002, 2004; Smith et al., 2004), from our EM single-label studies alone it was uncertain if the missing thalamic axodendritic input occurs for cholinergic interneurons or striatal projection neurons. In an EM double-label study to examine this, we found that cholinergic striatal interneurons in heterozygous Q140 mice, in particular, show a 40% deficiency in axodendritic thalamic input at 1 month of age (Deng et al., 2012). If a similar phenomenon occurs in homozygous Q140 mice (which seems likely), it may explain their reported hyperkinesia at 1 month, as the loss of thalamic input to cholinergic neurons would be predicted to more greatly lessen the responses of iSPNs than dSPNs to cortical drive (Smith et al., 2011), which models of basal ganglia function predict should cause hyperkinesia (Albin et al., 1989). The early rearing hyperkinesia at 2 months in homozygous Q94 knock-in mice (Menalled et al., 2002) and the open field hyperactivity at 3 months in heterozygous YAC128 mice (Slow et al., 2003), both before striatal projection neuron loss, might be explainable by this mechanism as well. An early deficiency in large axodendritic thalamic terminals on iSPNs, suggested by the present findings, may also contribute to the early hyperkinesia. Subsequent loss of cortical input to dSPNs during premanifest stages may lead to hypokinesia becoming the major motor manifestation.

### Thalamic input loss to striatal projection neurons in HD

Thalamostriatal projections end on the spines and dendrites of SPNs, with the proportion of spine vs. dendrite targeting being about 2:1 in rats and 4:1 in mice (Doig et al., 2010; Deng et al., 2013; Lei et al., 2013; Huerta-Ocampo et al., 2014). Thalamostriatal terminals are about half as abundant as corticostriatal terminals on the spines of SPNs, though more common on dendrites (Deng et al., 2013; Huerta-Ocampo et al., 2014). In general, dSPNs have been reported to receive a greater thalamic input than iSPNs in rats and monkeys (Sidibé and Smith, 1996; Lei et al., 2013). In the present study, we found a very similar abundance of VGLUT2+ terminals on D1+ vs. D1− spines and dendrites in 4 month− and 12 month-old WT mice, as have others (Doig et al., 2010; Huerta-Ocampo et al., 2014). Similarly, Wall et al. (2013) reported that dSPNs and iSPNs in mice receive input from relatively equal numbers of thalamic neurons. Nonetheless, the D1+ and D1− synaptic targets differ for WT mice in the shape of their size frequency distribution curves, with dSPN spines in 12-month old WT mice receiving synaptic contact from thalamic terminals with a size peak at 0.4 μm, and iSPN spines in 12-month old WT mice receiving synaptic input from populations of terminals with size peaks at 0.3–0.4 μm and 0.5–0.6 μm. This suggests that there may be thalamostriatal neuron subtypes that differ in their relative targeting of the two SPN types.

In any event, the present study shows that the deficiency in thalamic input to SPN spines that we previously demonstrated occurs early in the lifespan of Q140 mice (Deng et al., 2013) is largely comparable for dSPNs and iSPNs spines, and is not notably progressive from 4 to 12 months (except perhaps somewhat for D1− spines). Abnormalities in the part of thalamus projecting to striatum, such as increased GFAP expression, loss of the adhesion molecule tenascin-C, and loss of the synaptic protein complexin II, have been observed in other mouse models of HD (Kusakabe et al., 2001; Freeman and Morton, 2004). Given the thalamic atrophy and hypometabolism reported in premanifest HD, which eventually progresses to intralaminar thalamic neuron loss (Heinsen et al., 1999), an early deficiency in thalamic input to striatum may also occur in human HD victims (Paulsen et al., 2004; Feigin et al., 2007; Aylward et al., 2011). How this slight defect might affect behavior is uncertain. The absence of hypoactivity prior to 9 months in male Q140 heterozygous mice (Menalled and Howland, personal communication) suggests that the deficiency in thalamic input already seen at 1–4 months is not sufficient to cause motor hypoactivity. Similarly, hyperactivity has not been observed in male Q140 heterozygous mice (Rising et al., 2011) at 2.5 months of age or beyond, which suggests that the deficiency in thalamic but not cortical input already seen at 1–4 months could not by itself cause this motor abnormality either. Since thalamic input to striatum is thought to play a role in attentional mechanisms concerning motor planning and preparedness (Smith et al., 2004), more subtle behavioral tests may be required to detect the effect of the early deficiency in thalamic input to SPNs in Q140 heterozygous mice.

Of relevance to the basis of the thalamostriatal shortfall in Q140 mice, our observation that the deficiency in thalamic input does not notably progress from 4 to 12 months, except perhaps for iSPNs, and our prior finding that thalamostriatal input is already deficient at 1 month in Q140 mice raises the possibility that the deficiency involves an early developmental defect rather than a later pathological event. Consistent with this, striatal expression of proteins critical to thalamostriatal synapse formation, such as the semaphorin 3E receptor Plexin-D1 signaling complex, are significantly reduced early in the lifespan of several transgenic or knock-in HD mice (Kuhn et al., 2007; Ding et al., 2011), and in human HD as well (Strand et al., 2007).

### Regional pattern of cortical and thalamic terminal loss

We limited our analyses to dorsolateral motor striatum, and saw selective loss of presumptive IT-type corticostriatal axospinous terminals from dSPNs at 12 months, and relatively equal loss of thalamostriatal axospinous terminals from dSPNs and iSPNs already at 4 months. The striatum is, however, a heterogeneous structure that consists of distinct striosomal and matrix compartments. By analyzing dorsolateral striatum, we focused on matrix compartment SPNs and their motor function. As the organization of cortical and thalamic input to striosomes differs from that to matrix (Reiner et al., 2010; Crittenden and Graybiel, 2011), and as HD may differentially affect these two structures (Ferrante et al., 1987; Seto-Ohshima et al., 1988; Hedreen and Folstein, 1995; Deng et al., 2004; Crittenden and Graybiel, 2011; Waldvogel et al., 2012), we cannot know if the pattern of cortical and thalamic terminal loss we saw for dorsolateral matrix is also the case for striosomes. Moreover, the matrix compartment is regionally heterogeneous in terms of the parts of cortex and thalamus from which it receives input (Reiner et al., 2003, 2010; Crittenden and Graybiel, 2011). Thus, we cannot know if the pattern of cortical and thalamic terminal loss we saw for dorsolateral matrix compartment is the case for all of matrix. These issues will be of interest to address in future studies.

### Conflict of interest statement

The authors declare that the research was conducted in the absence of any commercial or financial relationships that could be construed as a potential conflict of interest.
